# Attitudes toward marriage among university students enrolled in a family counseling course: a comparative study

**DOI:** 10.1186/s40359-025-03343-2

**Published:** 2025-09-26

**Authors:** Rihan Thaher Altarawneh

**Affiliations:** Counseling and Mental Health Department, Faculty of Educational Sciences, The World Islamic Sciences & Education University W.I.S.E, Amman, 11947 Jordan

**Keywords:** Attitudes toward marriage, Family counseling course, University students, Jordan

## Abstract

**Background:**

Attitudes toward marriage (ATM) refers to an individual’s perceptions, and expectations of marital relationships, influenced by their cultural background, psychosocial factors, family of origin, and experiences in university life. This study aimed to validate the marital attitude scale (MAS) in Arabic by conducting validity and reliability tests. It also aimed to explore the levels of ATM among university students. Moreover, it compared two groups (group 1: enrolled in a family counseling course vs. group 2: did not enroll in a family counseling course) based on several demographic factors of gender, marital status, age, and type of school.

**Methods:**

This is a cross-sectional design among 450 university students in Amman-Jordan (102 participants enrolled in a family counseling course vs. 348 participants who did not enroll in this course). Data was collected from two public universities via online Google Forms from 10 September 2022 to 10 December 2022.

**Results:**

Results indicated that the Arabic version of MAS is valid and reliable by achieving all the criteria levels. More than 65% of Jordanian university students have a positive ATM. Students who enrolled in a family counseling course have better ATM than students who have not enrolled in this course (*p* < 0.001). Males and students attending humanistic schools are more likely to have positive ATM than female students and students attending scientific and medical schools.

**Conclusions:**

The findings could inform educational policy and encourage universities to include family counseling courses in the curriculum of different schools. Pre-marital counseling sessions should be implemented for prospective spouses to develop their competencies in managing life stressors effectively.

## Background

Attitudes toward marriage (ATM) refers to an individual’s beliefs, perceptions, and expectations of marital relationships, influenced by their cultural background, psychosocial factors, family of origin, and experiences in university life [1]. These aspects determine how individuals perceive marriage, their level of commitment, and their expectations toward marital roles. Among them, university life can prepare students for a positive ATM. Thus, educational courses may have a direct effect on attitudes and behaviors in their future marriage [2]. Policymakers and universities are responsible for integrating several educational courses that tackle couple and marriage counseling into students’ university lives.

Marital counseling sessions, whether conducted before or after marriage, have several benefits. It enhances marital satisfaction [3], improves the family value system [4], prevents marriage distress [5], and decreases divorce rates [6]. Families who seek counseling sessions in the late stages have serious relationship problems that lead to unresolvable conflicts [7]. Courses and sessions that tackle marriage and family counseling among university students can be effective in the success of marital life and psychological adaptation. Unfortunately, these courses are not mandatory among all university students, it is exclusive to students in schools of psychology, education, and sociology so far. In this regard, serious concerns and issues emerged in the academic final year, including suitable life partners, social relationships, financial issues, family of origin, and communication with partners [8]. Studying the effect of couple and family counseling courses on ATM among university students can enhance marital satisfaction and reduce the divorce rate.

The ATM is influenced by how individuals view marriage. It is affected by several factors including cultural norms, religious beliefs, economic conditions, and several demographic factors such as gender, region, age, marital status, and bearing children. A recent study explored the perspectives of married students toward marriage. The results showed that several beliefs, such as compassion, commitment, and conflict resolution, can create a healthy living atmosphere [9]. In 2024, a study was conducted and found that the female gender exhibited higher societal roles and lower martial attitudes than the male gender in Turkey [1]. In Egypt, females had more preparedness for couple and family counseling than males [10]. Unfortunately, illiterate women in lower-income countries were found to accept partner violence [11]. In Greece, almost 60% of university students prefer to bear two children only [12]. In the USA, university students who had positive ATM were found to be less engaged in cohabitation [13]. Studies that examine the differences between demographic factors and ATM in Arab countries, particularly among university students, are depleted. For instance, a study conducted in the United Arab Emirates focused on marital satisfaction among married people and found that men were more likely to be satisfied than women [14].

Several scales measure ATM, such as the Marital Attitudes and Expectations Scale (MAES), the Early Marriage Attitude Scale (EMAS), and the Marital Attitude Scale (MAS). The MAES (36 items) measures intent to marry as well as expectations for relationships [15]. The EMAS (40 items) measures several aspects among female adolescents, such as social norms and spiritual beliefs [16]. The MAS (23 items) is widely used and measures general satisfaction toward marriage [17]. Among them, the MAS has not been translated into Arabic and has fewer items; hence, we intend to translate it into the Arabic context.

In couple and family counseling courses, university students learn several subjects. For example, they learn the basic concepts of relationship systems, couple strategies, emotional connections, and dynamics of the marriage cycle [18, 19]. In addition to knowledge, it also teaches a variety of skills, including conflict resolution, communication skills, relationship coaching, and positive psychology interventions [20, 21]. This is the most significant issue addressed in our study, which hypothesized that university students who enrolled in these courses would have more positive ATM than those who did not enroll in these courses. Yet, few studies have assessed ATM among university students in the Arabic context.

### Study questions

1. Is the Arabic version of MAS valid and reliable among university students?
2. What is the level of ATM among university students?
3. Is there a difference between control and experimental groups toward ATM based on gender, marital status, age, and type of school?
4. Does a family counseling course have a direct effect on ATM among university students?

## Methods

### Study aim

This study aims to validate the MAS in the Arabic language by conducting a rigorous translation process, validity through face, content, and construct, and reliability through Cronbach’s alpha. It also explores the ATM among Jordanian university students. Furthermore, it investigates the differences between both student groups who enrolled in a family counseling course compared to students who have not enrolled in a family counseling course toward ATM based on demographic factors, including gender, marital status, age, and type of school.

### Study design

This is a cross-sectional methodological design to validate the MAS in the Arabic language. Also, we conducted a quasi-experimental design to explore the differences between both university student groups. This study followed both STROBE and TREND guidelines.

### Participants

The study population consisted of all university students in Amman, the capital of Jordan. We selected the two largest public universities in Amman. Our inclusion criteria were Amman City, bachelor’s degree, and different majors that taught a family counseling course. Universities in Amman that did not have a family counseling course were excluded. Moreover, diplomas, postgraduate degrees, and universities outside Amman City were excluded.

### Settings

We determined the sample size by using G*power software. Based on a medium-sized effect, p-value = 0.05, and power set at 0.80, the minimum required sample size was determined to be 150 participants. Moreover, a previous international study highlighted 150 participants for the main analysis with a moderate effect size and an alpha value of 0.05 [22]. However, this study employed a convenient sampling method to recruit participants from two public universities in Amman-Jordan. However, we created an online survey using Google Forms containing a written informed consent by ticking the box (agree to participate in this study: yes, no), demographic information (gender, age, type of school, and marital status), and MAS. The cover letter in the Google Form assured them that they had the right to participate and withdraw at any time, and we assured them that their information would only be used for research purposes.

We conveniently selected the two largest public universities in Amman according to our inclusion criteria. We distributed an online survey through official WhatsApp groups and via faculty members who had access to university students between 10 September 2022 and 10 December 2022, depending on the snowball procedure. To prevent duplicate responses from the same school student, we asked them to provide their school ID. By visiting the three largest schools: medical, scientific, and humanities, we ensured broad coverage of the university’s student population. Moreover, faculty members in each school were approached in person to assist in distributing the online questionnaire, ensuring that all students had an equal opportunity to participate in the study. A priori power analysis indicated that a minimum of 150 participants was required to achieve adequate statistical power. Data were collected from a total of 450 university students. This number exceeds the required sample size and represents the study sample. The online survey took almost 3 min to complete.

### Group assignment and experimental design

From the total pool of respondents (*n* = 450), we identified participants based on whether they had completed a university-level course in family counseling by asking a direct question: “Did you receive a family counseling course?” Of the total respondents, 102 students (mainly from the School of Humanities) had previously completed a family counseling course and were thus assigned to the experimental group. The remaining 348 students, who had not taken the course, comprised the control group. While the study did not use randomized group assignments, we attempted to control confounding variables by ensuring similar distribution of gender, age, marital status, and academic major between groups. These groups were matched as closely as possible to evaluate the potential influence of that exposure on ATM. Participants were represented from three academic sectors (humanities, medical, and scientific), allowing for representation across diverse academic backgrounds.

For the sample size in each group, it was determined that a minimum of 50 participants is required for a simple comparison of two within-participant conditions when aiming for 80% statistical power [23]. A subsample of 40 participants was randomly selected from each group. The control group (*N* = 40) comprised students who had not enrolled in the family counseling course, whereas the experimental group (*N* = 40) included students who had completed the course. A flowchart 1. illustrates the comparison between the two groups.

**Flowchart 1:**
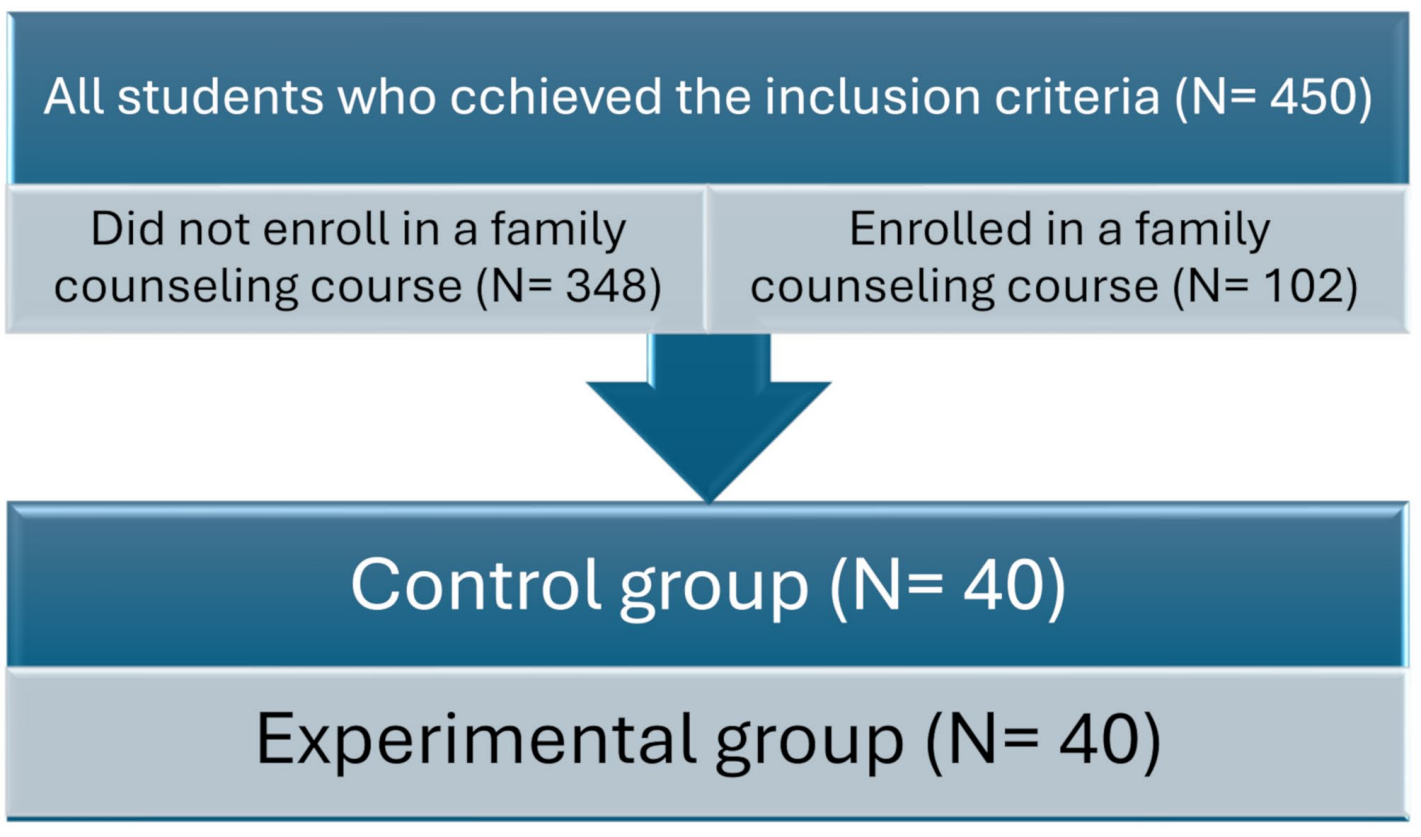
Comparison between students who enrolled in a family counseling course and students who did not

### Study tool

#### Demographic information

We included gender (male, female), age (below 20 years, 20–23 years, type of school (medical, scientific, humanities), and marital status (single, married, others).

#### Marital attitude scale

This study used MAS, which consists of 23 items. It is a widely known scale and measures general satisfaction toward marriage [17]. This scale is open-access and can be used by researchers without official permission. Participants responded to MAS through a four-point Likert scale ranging from strongly agree 0 to strongly disagree 3. Item numbers (1, 3, 5, 8, 12, 16, 19, and 23) were rated in reverse order. The higher the scores, the higher the positive ATM. Students who have mean scores ≤ 1 indicate a low attitude toward marriage, those with scores between 1 and 1.99 indicate an average level, and those with scores greater than 2 indicate a high attitude toward marriage.

#### Family counseling course

This course, offered over one semester for university students, explores various couple and family theories, challenges, and intervention strategies related to couples and families. It also focuses on addressing family dynamics, conflict resolution, and effective communication approaches to managing and resolving issues within the family unit as a whole.

### Statistical analysis

Data were entered into SPSS v.25 for analysis. First of all, we cleaned the data from outliers, then we checked normality with the Kolmogorov-Smirnov test. To test the validity and reliability of MAS in the Arabic language, we used Lawshe’s content validity ratio (CVR) Table, content validity index (CVI), principal component analysis (> 0.30), Eigenvalues (> 1.5), Kaiser-Meyer-Olken (KMO > 0.70), Bartlett’s test of sphericity (*p* < 0.05), and Cronbach’s alpha value (> 0.70) [24, 25]. We performed descriptive statistics, including means, standard deviations (M ± SD), and frequencies, to measure ATM levels among university students. Multivariate Analysis of Covariance (MANCOVA) using a factorial design (1 × 2) was used to compare the differences between experimental and control groups. The two-way analysis of variance (ANOVA) was also used among experimental and control groups based on demographic factors including gender, age, type of school, and marital status. An eta-squared (η²) analysis was used to determine the effect size, assessing the effectiveness of a family counseling course and the proportion of explained variance in the dependent variable. Based on the guidelines, 0.01 indicates a small effect, 0.06 indicates a medium effect, and 0.14 indicates a high effect [26]. Linear regression analysis was performed to explore the effect of a family counseling course on ATM among university students. The p-value was set at 0.05.

### Ethical considerations

Ethical approval was sought from the World Islamic Sciences and Education University (No: 5/1/9/781) before collecting the data from participants. A written informed consent from all participants was sought by ticking the box in Google Form (agree to participate in this study: yes, no). Ethical principles and guidelines were followed according to the Declaration of Helsinki 1964. Participants have the right to participate and withdraw at any time, and their information will be used for research purposes.

## Results

### Psychometric properties of MAS

We hypothesized that MAS is valid and reliable among university students by conducting a translation process, validation, and reliability as follows.

#### Translation process

The MAS has been translated into Arabic by experts in Arabic-English translation and specialized experts in psychology and counseling. We requested that the English version be translated into Arabic, and then other experts requested that the Arabic version be translated into English. Comparing the translation process, the final 23 items in the Arabic version are consistent and meet the standards.

#### Face validity

To ensure the face validity of MAS, it was presented in Arabic form to 30 students who were selected from outside the study sample. We requested that students provide suggestions regarding the clarity, simplicity, and ease of understanding of the items. All students indicated that the Arabic MAS has achieved the criteria levels.

#### Content validity

The Arabic MAS was presented to 10 experts who specialized in counseling, mental health, and psychology from various Jordanian universities. We rated the items using a four-point Likert scale regarding relevancy. The CVI for each item of the Arabic MAS surpassed 92%. Lawshe’s CVR indicated an agreement of a minimum of 62%, thus, based on expert feedback, nine out of ten experts approved the Arabic MAS items as they were achieving a 90% agreement rate. Minor modifications were made to certain items based on their recommendations.

#### Construct validity

**Fig. 2:**
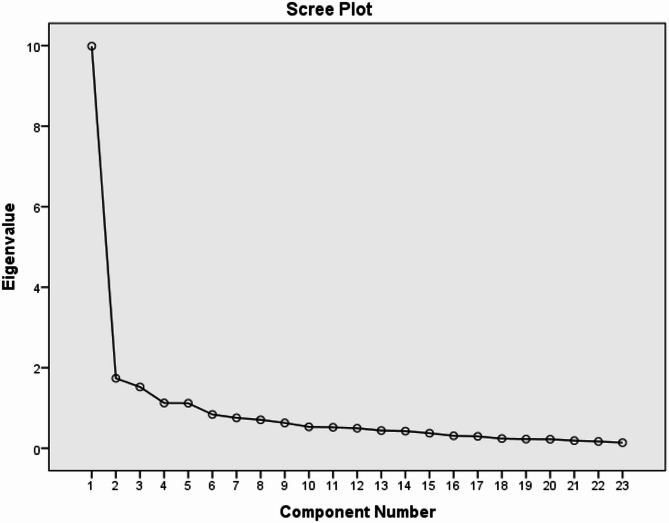
Scree plot for the Arabic MAS

The correlation coefficients of the Arabic MAS items ranged between 0.44 and 0.89. The KMO was 0.90. Bartlett’s test of sphericity was significant (Chi-Square: 1762.7, *p* < 0.001). The scree plot Fig. 2 shows three constructs loading with an eigenvalue above 1.5. The total cumulative variance was 57.60% for the Arabic MAS. All values confirm the construct validity for the Arabic MAS.

#### Reliability

The Cronbach’s alpha coefficient was calculated for the Arabic MAS, yielding a value of 0.94. These results indicate that the Arabic version of the MAS demonstrated acceptable levels of validity and reliability, with a satisfactory model fit.

### Demographic information

The total number of university students participating in this study was 450. Our data were normally distributed (*p* > 0.001). More than half of the students were female, single, aged between 20 and 23 years old, and studied in humanities schools such as counseling, psychology, and special education (Table 1). More than two-thirds (77.3%) of university students did not enroll in a family counseling course.

**Table 1:** Demographic characteristics (*N* = 450)

Variables	Descriptive	Frequency (%)
Gender	Male	166 (36.8)
	Female	284 (63.2)
Marital status	Single	371 (82.4)
	Married	71 (15.7)
	Others	8 (1.9)
Age (years)	< 20 years	206 (45.8)
	20–23 years	244 (54.2)
Type of school	Medical	76 (16.9)
	Scientific	116 (25.8)
	Humanities	258 (57.3)
Did you receive a family counseling course	Yes	102 (22.7)
	No	348 (77.3)

### Attitudes toward marriage levels

We calculated the overall score of the ATM among all (450) students. We found that they had an average score (1.96 ± 0.65) which equals a percentage of 65.33%. After randomly selecting the control (did not enroll in a family counseling course) and experimental groups (enrolled in a family counseling course), we found that the control group (1.42 ± 0.44; 47.33%) had a lower mean and percentage score than the experimental group (2.10 ± 0.56; 70%) for the ATM. Moreover, we found a significant difference (*p* < 0.001) between the control and experimental groups after conducting an ANCOVA test, and Table 2 shows the result.

**Table 2:** ANCOVA results for the differences between control (*N* = 40) and experimental groups (*N* = 40)

Source of variance	Sum of squares	DF	Mean square	F-value	*p*-value	Eta-squared (η²)
Pretest (covariate)	34.894	1	34.894	3.550	0.070	—
Training course	5768.533	1	5768.533	586.91	0.001***	0.77
Error	265.372	37	9.829	—	—	
Adjusted Total	6068.800	39	—	—	—	

Note: DF: degree of freedom, *p < 0.05, **p < 0.01, ***p < 0.001

The effect size was calculated using Eta-Squared (η²), yielding a value of 0.77. This indicates that the family counseling course explained 77% of the total variance in increasing ATM among university students.

### Differences between control and experimental groups based on demographic factors

We hypothesized that there is a difference between control and experimental groups toward ATM based on gender, marital status, age, and type of school. By running a two-way ANOVA test, we found that male students (f = 2.84, *p* = 0.005, η²= 0.08) exhibited higher levels of ATM than female students (Table 3). Moreover, the type of school (f = 38.29, *p* < 0.001, η²= 0.10) was found to be associated with ATM. Students in humanistic schools revealed higher ATM than other school types. There were no differences in marital status (f = 0.97, *p* = 0.33) and age (f = 1.02, *p* = 0.31) in ATM based on control and experimental groups.

**Table 3:** Comparison between control (*n* = 40) and experimental groups (*n* = 40) based on demographic factors

Variables	Descriptive	Control	Experimental	f-value	*p*-value	Eta square
Gender	Male	15 (37.5)	14 (35)	2.84	0.005**	0.08
	Female	25 (62.5)	26 (65)			
Marital status	Single	34 (85)	27 (67.5)	0.97	0.33	0.009
	Married	6 (15)	13 (32.5)			
Age (years)	< 20 years	26 (65)	19 (47.5)	1.02	0.31	0.01
	20–23 years	14 (35)	21 (52.5)			
Type of school	Medical	6 (15)	5 (12.5)	38.92	0.001***	0.10
	Scientific	16 (40)	15 (37.5)			
	Humanities	18 (45)	20 (50)			

Note: M ± SD: Mean ± Standard deviation, *p < 0.05, **p < 0.01, ***p < 0.001

### Effect of a family counseling course on ATM

We hypothesized that a family counseling course has a direct on ATM. Conducting a simple linear regression model on all university students (450), we found that a family counseling course had a direct effect on ATM (f = 88.069, *p* < 0.001), explaining 35% of the total variance (Table 4).

**Table 4:** Effect of a family counseling course on students’ attitudes toward marriage (*N* = 450)

*R* (Correlation Coefficient)	*R*² (Coefficient of Determination)	Adjusted *R*²	F-value		*p*-value	
0.592	0.35	0.35	88.069		0.001***	
Independent Variable		Regression Coefficient (B)	Standard Error	Beta	Calculated t-value	*p*-value
Family counseling course		0.65	0.12	0.59	5.24	0.001***

Note: Dummy variables 1: enroll in the course (*N* = 102), 0: did not enroll (*N* = 348), ***p < 0.001

## Discussion

The result highlights that the MAS in the Arabic language is valid and reliable for measuring ATM among university students. It highlights also that a family counseling course has a positive ATM on university students. Male students have more positive ATM than female students. Students in humanistic schools are more likely to have positive ATM than students in scientific and medical schools. Family counseling courses have a significant effect on increasing students’ marriage attitudes and satisfaction. These results are consistent with previous studies [3, 14, 17].

To our knowledge, this is the first study in the Arabic context to examine the validity and reliability of MAS. Compared to previous scales such as the MAES [15] and EMAS [16], the MAS is more comprehensive, more culturally relevant, and has fewer items. After a rigorous translation and back-translation process from specialized experts and after assessing face, content, construct validity, and internal consistency, we found that the MAS is valid and reliable among university students. Nine out of ten experts agreed on the final version of the Arabic MAS. During construct validity, we found that all items loaded above 0.44, which is higher than what was previously reported in the original scale [17]. The Arabic MAS revealed three factors that accounted for 57.60%. Comparing our results with previous studies, it disagrees with the original MAS, which is loaded with one factor and agrees with the general attitudes toward marriage scale, which has 10 items and is loaded with three factors, namely positive attitudes, negative attitudes, and affective reaction [27]. Our finding is consistent with the Persian MAS version, which showed that Persian MAS is valid and reliable among 137 university students [28]. The Cronbach alpha value in this study is higher than what was reported on the original scale of 0.85. It is worth mentioning that other scales have been used to measure the intent to marry scale [29], love and respect scale [30], and marital love scale [31]. The Arabic MAS will help Arabic researchers to apply it in different areas such as universities, courts, and organizations.

Overall, more than 65% of Jordanian university students have a positive ATM, a result that is consistent with the findings of a previous survey among 820 midwifery and nursing students in a university in Turkey [32]. In contrast, students who took the family counseling course have a better attitude toward marriage than students who did not receive this course. This may be related to the fact that the course material provides conflict resolution strategies, communication skills, active listening, understanding of the emotional needs of the partner, and therefore a healthy relationship with the partner. Additionally, it provides information about romantic attachment style, which plays a crucial role in marital satisfaction [33]. Several studies have focused on the effects of marriage counseling sessions on marital satisfaction [34], intimacy [35], and emotion regulation [36]. We found that university students who enrolled in a family counseling course had more positive attitudes than students who did not. Comparing our results with previous studies. In Iran, they conducted a study and found that premarital education leads to more marital satisfaction than participants who did not enroll in such education [37]. Similarly, a study found in Los Angeles County that premarital education empowers couples and maintains their relationship [38]. A review study found that any type of couple-based therapy and treatment improves relationships and reduces distress among couples [39]. Therefore, we recommend that couple and family counseling courses become mandatory for all university students across different schools.

Males and students attending humanistic schools have more positive ATM than females and other schools. This can be attributed to social and cultural factors. For instance, men are generally expected to seek out their partners and may receive social benefits from marriage and a normal life cycle. Compared to other schools that focus on personal achievement and career development over marriage, students in humanistic schools are more concerned with human resilience behavior, beliefs, and values, making them more positive toward marriage [40]. Our findings disagree with previous studies, which found no differences between genders in their ATM [41], while another study found that females have more intentions, desires, and attitudes to marry than young males [42]. Medical professions have more challenges and barriers than other professions [43, 44], but when they are married, they have higher satisfaction levels [45] and lower divorce rates [46]. More studies are warranted regarding this issue.

### Limitations and future studies

We acknowledge that using a cross-sectional design, a convenience sampling method, and data collection restricted to a single geographic area within Amman City may limit the generalizability of our results. Moreover, we acknowledge that the use of non-randomized group assignment represents a methodological limitation of the study. Future research should explore the impact of additional demographic variables and other cities to enhance the generalizability of findings. Conducting more relationships with ATM such as life satisfaction, meaning in life, and resilience are needed in future studies. The Arabic version of MAS should be applied to other Arab countries with different sample characteristics to discover its applicability.

## Conclusion and recommendation

The Arabic version of MAS exhibits validity and reliability among university students. The translation process followed a rigorous methodology to ensure linguistic and conceptual accuracy. Factor analysis results yielded significant results. More than 65% of Jordanian university students have positive ATM. Students who enrolled in a family counseling course have better ATM than students who have not enrolled in this course. Males and students attending humanistic schools are more likely to have positive ATM than female students and students attending scientific and medical schools. These courses and sessions are designed to tackle the rising rates of divorce and marital dissatisfaction. The findings could inform educational policy and encourage universities to include family counseling courses in the curriculum of different schools to better support university students in their future lives. Additionally, prospective spouses should participate in pre-marital counseling sessions and courses to develop their competencies for managing life stressors effectively. There is a need for more proactive study on ATM, including longitudinal studies, work-life balance, and parental marital status.

## Data Availability

The data that support the findings of this study are not openly available due to reasons of sensitivity and are available from the corresponding author upon reasonable request. Data are located in controlled access data storage at World Islamic Sciences and Education University (deewan.education@wise.edu.jo, phone #: (00962 (6) 560 0230).

## Abbreviations

ATM: Attitudes toward marriage
CVR: Content validity ratio
CVI: Content validity index
KMO: Kaiser-Meyer-Olkin
